# Comprehensive Context Recognizer Based on Multimodal Sensors in a Smartphone

**DOI:** 10.3390/s120912588

**Published:** 2012-09-17

**Authors:** Manhyung Han, La The Vinh, Young-Koo Lee, Sungyoung Lee

**Affiliations:** Department of Computer Engineering, Kyung Hee University (Global Campus), 1 Seocheon-dong, Giheung-gu, Yongin-si, Gyeonggi-do 446-701, Korea; E-Mails: smiley@oslab.khu.ac.kr (M.H.); vinhlt@oslab.khu.ac.kr (L.T.V.); yklee@khu.ac.kr (Y.-K.L.)

**Keywords:** context aware, smartphone, context recognition, accelerometer classification, audio classification, multimodal sensors

## Abstract

Recent developments in smartphones have increased the processing capabilities and equipped these devices with a number of built-in multimodal sensors, including accelerometers, gyroscopes, GPS interfaces, Wi-Fi access, and proximity sensors. Despite the fact that numerous studies have investigated the development of user-context aware applications using smartphones, these applications are currently only able to recognize simple contexts using a single type of sensor. Therefore, in this work, we introduce a comprehensive approach for context aware applications that utilizes the multimodal sensors in smartphones. The proposed system is not only able to recognize different kinds of contexts with high accuracy, but it is also able to optimize the power consumption since power-hungry sensors can be activated or deactivated at appropriate times. Additionally, the system is able to recognize activities wherever the smartphone is on a human's body, even when the user is using the phone to make a phone call, manipulate applications, play games, or listen to music. Furthermore, we also present a novel feature selection algorithm for the accelerometer classification module. The proposed feature selection algorithm helps select good features and eliminates bad features, thereby improving the overall accuracy of the accelerometer classifier. Experimental results show that the proposed system can classify eight activities with an accuracy of 92.43%.

## 1. Introduction

Context recognition is a highly active research area due to its large number of potential applications such as in healthcare, virtual reality, security, surveillance, and advanced user interface systems. As a result, it has caught the attention of researchers from industry, academia, security agencies, consumer agencies, and even the general populace. Several years ago, such context aware systems were mostly based on complicated wearable sensors, which are not even commercially available nowadays. However, the recent, rapid development of the smartphone industry has enabled implementation of context aware applications using the large number of sensors already integrated within smartphones [1,2].

Nevertheless, substantial progress has only been made for recognition of simple user contexts using a single type of sensor, such as the accelerometer [3], GPS [4], or audio tool [5]. Although some recognition of user contexts may be possible with particular sensors, such an approach is not able to support a comprehensive and realistic context aware device. For example, to merely recognize ambulatory contexts like walking or jogging, the accelerometer or gyroscope achieves a reasonable accuracy [6,7]. Likewise, to classify acoustic contexts, such as in a bus, subway, or meeting place, the audio data can be utilized [8]. The GPS has also been used as a single source to classify different contexts [4,9,10]. Yet, a comprehensive recognition system should make use of all those sensors in order to be capable of recognizing a higher number of mixed contexts including ambulatory, transportation, and acoustic. Furthermore, the use of multiple sensors can improve the power consumption since some sensors can then be activated only when necessary. For example, a system that recognizes transportation by inferring the user's GPS route [11] can stop collecting GPS data if an accelerometer classifier detects that the user is walking.

Motivated by the lack of a comprehensive approach in smartphone-based context recognition research, we propose a multimodal context recognizer utilizing several kinds of sensors in a smartphone. We also consider that the activity recognition must be performed regardless of what the user is doing with his or her smartphone, such as making a phone call, using applications, playing games, or listening to music. Thus, we propose a position-free recognition system that recognizes a human's activities wherever the smartphone is attached on the body. It provides high degree of freedom to users, as well as ample practical relevance.

Besides the classification aspect, the proposed system pursues the optimal combination of sensors in order to reduce the power consumption, which is a vital issue for any smartphone application [12]. The system utilizes the accelerometer to detect transition points from ambulatory activities to transportation activities and *vice versa*. The audio classifier is only activated if there is a further need to classify transportation activities, such as using a bus or subway. By using the above approach, we can save power on smartphone devices.

**Figure 1.**
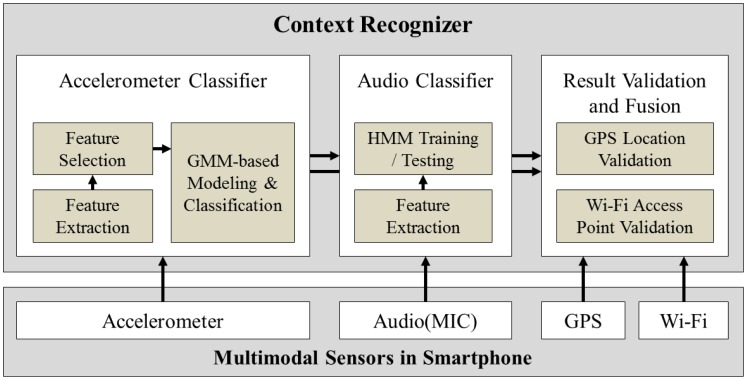
Overall architecture of the proposed system—Context Recognizer.

Finally, the proposed system combines and validates the output of the two classifiers using extra information from the GPS and Wi-Fi functions to produce the final result. By following this approach, the system is able to classify both ambulatory as well as transportation contexts, while still achieving low power consumption. The overall architecture of the proposed solution is presented in Figure 1.

As described in Figure 1, for the overall architecture, we used Gaussian Mixture Model (GMM) for the acceleration data classification and Hidden Markov Model (HMM) for the audio classification. Before modeling and classifying acceleration data, a prior process including feature extraction and selection generates bunch of features to be used for a classification. In order to use multiple dimensions of features, mixture model which is suitable for representing multiple distributions of collected data is chosen. Other classification techniques such as Gaussian Process are more appropriate for considering small number of variables or features. For the audio classification, we used HMM algorithm for training and testing audio data because the module needs to be classify only two activities—bus and subway—and requires running on a smartphone in real-time. There are other audio classification algorithms such as Conditional Random Field and Support Vector Machine, but our approach using HMM is lighter than other algorithms and it fits in classifying similar audio data both collected from bus and subway.

## 2. Related Works

The high availability of smartphones with built-in sensors (accelerometer, gyroscope, GPS, Wi-Fi, *etc.*) is highly advantageous to the research area of context recognition. In [3,6,7], a smartphone accelerometer was used to recognize user movement contexts such as walking and running; in [5,8], the author utilized audio data to classify acoustic environments. The authors of [4,9,11] showed that GPS can be used to recognize transportation routines. However, we must note that those works merely exploited a particular sensor instead of combining the strength of multiple sensors. To the best of our knowledge, [2] is one of the first works to combine accelerometer and audio classification; the author demonstrated that the combination of audio helps improve the accuracy of recognizing user activities.

In [13], the authors designed and implemented both an audio classifier and accelerometer classifier using audio and accelerometer sensors. The modules are similar to our work but the approaches to recognize contexts are different. In their system, each classifier can recognize only one specific context—the accelerometer classifier recognizes human behaviors such as sitting, standing, walking and running, on the other hand, the audio classifier's purpose is to determine whether a person is in a conversation or not—but our proposed system utilizes both classifiers and other sensors together for classifying contexts as described in Figure 1.

More recently, [11] is the most similar to our work in that the author classified the mobile acceleration in order to detect whether a user was riding a transit vehicle, after which his system activated the GPS recorder and matched the GPS route to identify different types of transportation. Unfortunately, route matching may necessitate the collection of a long duration of data, meaning that the system cannot respond in real-time. Moreover, an extensive collection of GPS data can deplete the phone battery. Accordingly, in our work, we propose the use of audio to differentiate between various types of transportation since only a few seconds of audio recording is necessary for this purpose. Consequently, using audio not only reduces the system response time, but also improves the battery power consumption.

For accelerometer classification methods, there are a large number of proposed solutions [14] with assorted feature extraction techniques and classification algorithms. In [3], which is one of the most cited papers in the area of accelerometer-based activity recognition, the author proposed using frequency domain features in combination with a decision tree classifier; this approach yielded good results and has since been supported by other published works [7,14]. However, in other papers, such as [6,15], the authors noted that there are other accelerometer features that may produce even better results. While the field has not reached a final agreement on the topic of feature extraction for accelerometer signals, we have selected a hybrid approach by proposing our own feature selection algorithms [16]. Therefore, instead of using predefined features obtained via a particular type of feature extraction technique, we have utilized several potential approaches, after which our feature selection algorithm will be executed to select the best features from the whole set.

In the research area of feature selection, the numerous proposed solutions can be generally categorized into three main directions: wrapper [17,18], filter [19] and embedded [20]. The performance of the wrapper and embedded directions depends strongly on the classifier used in the selection process. In addition, repeatedly training and evaluating the classifier in order to select features results in a very high execution cost. The filter method, on the other hand, utilizes a simple classifier-independent measurement to judge the quality of the features, thereby allowing it to work with different classifiers and requiring less time to execute the selection process. Nevertheless, recently published works regarding filter-based feature selection methods, such as [21,22] still cannot completely overcome the notorious challenge of balancing the relevance and the redundancy. In this work, we propose a filter feature selection method to overcome that limitation, as well as to improve the classification accuracy.

In audio classification research, the authors in [5] highlighted that audio recordings were a rich source of information that could be used to recognize contexts. In their work, they employed the well-known audio feature, Mel Frequency Cepstral Coefficients (MFCCs), in combination with the hidden Markov model (HMM) classification algorithm. However, it should be noted that the audio in their work was recorded with an external microphone, which often has a higher quality than a mobile phone's built-in microphone, and was processed offline. Recently, the author of [8] proposed a scalable sound sensing system, which was implemented on an iPhone and was shown to work well in realistic environments.

## 3. The Proposed System

**Figure 2.**
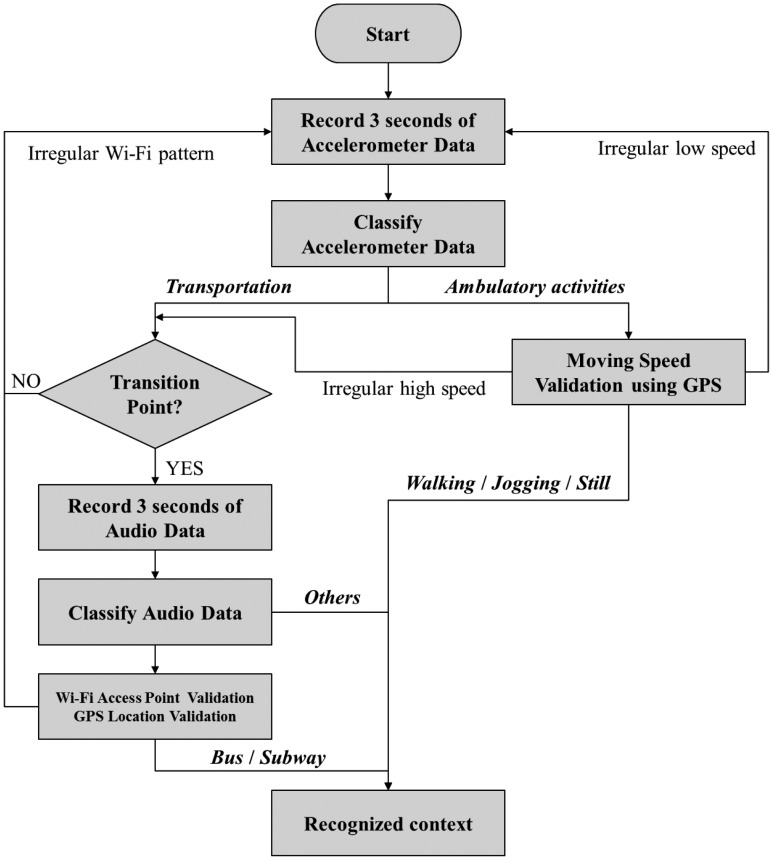
Flow chart of the proposed system.

As can be seen from Figure 2, our system starts by recording three seconds of accelerometer data and then classifying those data into two categories:

- Ambulatory activities—Walking, Jogging or Still
- Transportation

For classifying ambulatory activities and transportation, we first utilize acceleration data from accelerometer. If collected data has regular pattern such as walking, jogging and still, the system classify it as an ambulatory activity, but if it shows an irregular pattern, the output is regarded as transportation. If the output is ‘ambulatory activities’, the system determines whether the user is walking or jogging at a reasonable speed based on the speed information from the GPS interface. If the speed is reasonable or if a GPS signal is not available, the system outputs the final recognized context. Occasionally, a running bus may be misrecognized as “walking” or “jogging,” and in such a case, the speed validator will redirect the next processing step to the ‘transportation’ branch. In the ‘transportation’ branch, the system first determines whether a transition point occurred (*i.e.*, the previous recognized context was not ‘transportation’). Then, if a transition point did occur, the audio recorder will activate to record another three seconds of audio data. The system will then classify these three seconds of sound into three categories:

- Bus
- Subway
- *Others* (all other sound that is not a bus or a subway)

The result of the audio classifier can be further validated using a Wi-Fi pattern. More specifically, subway systems possess only a small number of well-known Wi-Fi services, and private wireless networks are nearly non-existent inside subways. In contrast, buses run on streets where private wireless networks from the passing buildings are abundant and often appear in the user range only to disappear a short time later. Consequently, these different Wi-Fi patterns can be used to validate the result of the audio classifier and avoid ambiguity in recognizing a bus and a subway.

Further validation can be done through the use of GPS readings, if available. For example, we prerecorded the locations of all the subway stations in Seoul, which totaled around 100 stations. Hence, if a user approaches a subway, his latest location should be near a station (*i.e.*, within a radius of 200 m). In short, the proposed system makes use of several sensors, including the accelerometer, audio tool, GPS, and Wi-Fi, and is able to recognize at least five different contexts:

- User is walking
- User is jogging
- User is riding a bus
- User is riding a subway
- Other contexts (the context that is not one of the above four target contexts)

The system mainly employs the accelerometer and audio recordings to classify the contexts. It uses extra information from the GPS and Wi-Fi systems to validate the results of the classification modules.

### 3.1. Accelerometer Classification

#### Feature Extraction

In our system, instead of using a single method, we utilize several kinds of well-known feature extraction techniques to construct a high number of features; then we select the best features using our own feature selection algorithms. We consider the following features:

- Time domain features: standard deviation, mean crossing rate, Pearson correlation coefficients
- Frequency domain features [3]
- Linear Predictive Coding (LPC) features [15]

#### Feature Selection

Since we have a large number of features, using all of them may not increase the accuracy due to the problem known as ‘the curse of dimensionality’. Consequently, it is necessary to select the best features from the extracted ones in order to construct a good feature set. Our proposed method [16] measures the quality of a feature based on two criteria: the relevancy of the feature (or the classification power) and the redundancy of the feature (or the similarity between two selected features). These two criteria are computed from the mutual information of the feature as described in Equations (1) and (3):

(1) $$\text{Rel}(X)=\frac{I(C;X)}{log_{2}(\left|\Omega_{C}\right|)}$$

where *X* is a feature variable, *C* is a class variable, and Ω*_C_* is the state space of *C*. Note that *I*(*C;X*) is the mutual information between *C* and *X*, which can be calculated by:

(2) $$I(C;X)=\sum_{c\in \Omega_{C}}\sum_{x\in \Omega_{X}}p(c,x)log_{2}\left(\frac{p(c,x)}{p(c)p(x)}\right)$$

where Ω*_X_* is the state space of the variable *X; p*(*c*, *x*), *p*(*c*), and *p*(*x*) are, respectively, the joint and marginal probability distributions:

(3) $$\text{Red}(X,Y)=\frac{I(X;Y)}{log_{2}(\left|\Omega_{X}\right|)}$$

**Algorithm 1**. Feature Quantization.
1:**Input:***M* – Total number of features2:*X*(1..*M*) – Training data3:*Δ* – The quantization error4:**Output:***N* – Number of quantization levels5:*Y*(1..*M*) – Quantized data6:**Quantization**7: *N* = 2;8: **while** 1 **do**9:  *MaxError* = −1*e*+16;10:  **for**
*m* = 1 to *M*
**do**11:   *Upper* = *max*(*X*(*m*));12:   *Lower* = *min*(*X*(*m*));13:   *Step* = (*Upper* – *Lower*) / *N*;14:   *Partition* = [*Lower : Step : Upper*];15:   *CodeBook* = [*Lower* – *Step*, *Lower : Step : Upper*];16:    [*Y*(*m*), *QError*] = *Quantiz*(*X*(*m*), *Partition*, *CodeBook*);17:   **if**
*QError* > *MaxError*
**then**18:    *MaxError* = *QError*;19:   **end if**20:  **end for**21:  **if**
*MaxError* < *Δ*
**then**22:   **break**;23:  **end if**24:  *N* = *N* + 1;25: **end while**26:**end Quantization**

**Algorithm 2**. Greedy Forward Searching for Feature Selection.
1:**Input:***M* – Total number of features2:*N* – Total number of data samples3:*K* – Number of features to be selected4:*X* – Training data matrix (*M × N*)5:*C* – Class labels (1 × *N*)6:**Output:***S* – The index vector of the selected features (1 × *K*)7:**Forward**8: *S* = *Φ*;9: **for**
*m* = 1 to *M* d**o**10:  *X_m_* = X_m_ − *μ*(*X_m_*);11:  *X_m_* = *X_m_* / *σ*(*X_m_*);12: **end for**13: *X* = *Quantiz*(*X*);14: **for**
*k* = 1 to *K*
**do**15:  *BestScore* = −1*e*+16;16:  *BestIndex* = 0;17:  **for**
*i* = 1 to *M*
**do**18:   **if**
*X_i_* not in *S*
**then**19:    *f* = 0; *c* = 0;20:    **for**
*X_j_* in *S*
**do**21:     *c* = *c* + 1; *f* = *f* + *Red*(*X_i_*, *X_j_*);22:    **end for**23:    *f* = *Rel*(*X_i_*) – *f*/*c*;24:    **if** (*f* > *BestScore*) **then**25:     *BestScore* = *f*;26:     *BestIndex* = *i*;27:    **end if**28:   **end if**29:  **end for**30:  *S* = {*S*, *BestIndex*};31: **end for**32:**end Forward**


In the above Equations (1) and (3), the mutual information can be computed by summing over the state space of the variable; therefore, the variables should be discretized before such a calculation can be performed. The discretization algorithm is illustrated in Algorithm 1. Once the relevance and the redundancy have been computed, we can utilize the well-known searching mechanism called ‘greedy forwarding’ to gradually extend the selection of features. The whole selection process is illustrated in Algorithm 2.

#### Gaussian Mixture Classifier

After extracting and selecting features, let us assume that *X^C^* is a training data matrix (*N* × *K*) for class C, where each row is a training sample, and each column is a feature value. We utilize a Gaussian mixture model (GMM) to determine the parametric probability density function of each class, denoted by *p*(*X^C^*∣*λ^C^*), where *λ^C^* is the parameter set that includes the mixing weights and individual Gaussian mean vectors and covariance matrices:

(4) $$p(X^{C}\;|\;\lambda^{C})=\sum_{i=1}^{M}\omega_{i}N(X^{C}\;|\;\mu_{i},\sum_{i})$$

where *N* is a Gaussian distribution and is given by:

(5) $$N(x\;|\;\mu_{i},\sum_{i})=\frac{1}{{\left(2\pi \right)}^{D/2}{\left|\sum_{i}\right|}^{1/2}}\text{exp}\left\{-\frac{1}{2}{\left(x-\mu_{i}\right)}^{\prime }{\sum_{i}}^{-1}(x-\mu_{i})\right\}$$

The mixing weights must satisfy the following condition:

(6) $$\sum_{i=1}^{M}\omega_{i}=1$$

During the training phase, the parameters *λ^C^* = {*ω, μ, Σ*} are determined to maximize the training data likelihood *p*(*X^C^*∣*λ^C^*). In the inference phase, given all the class parameter sets λ^C1^, λ^C2^, …, λ^Cm^ and an input vector *x*, the class label is determined by:

(7) $$C=\text{argma}x_{C}(p(x\;|\;\lambda^{C}))$$

### 3.2. Audio Classification

**Figure 3.**
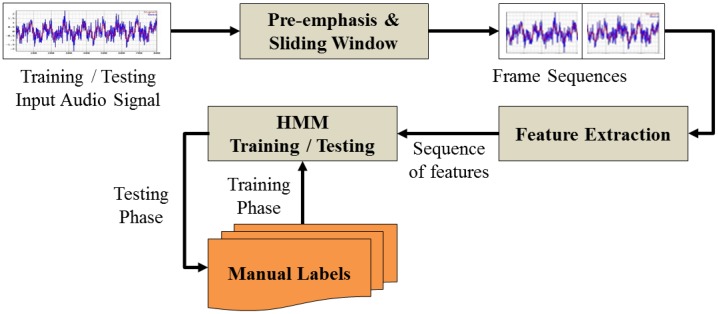
System architecture of the audio classification module.

For the audio classification module, we combine MFCCs [5] at frame level with the conventional classification method using the hidden Markov model. Figure 3 illustrates the audio classification module.

#### 3.2.1. MFCC Feature Extraction

Before the computation of MFCCs, a pre-emphasis filter is applied to the input audio signal *x*(*n*), which eliminates the high frequencies:

(8) x(n)=x(n)−0.9x(n−1)

Next, the filtered signal is divided into shorter frames and multiplied with a Hamming window function such that:

(9) $$w(n)=0.54-0.46\cos\left(\frac{2n\pi }{N-1}\right)$$

(10) y(n)=w(n)x(n)

where *N* is the length of a window.

The feature extraction component then transforms the signal frames into the frequency domain using a discrete Fourier transform (DFT):

(11) S(n)=DFT[y(n)]=R(n)+jI(n)

(12) $$P(n)=\left|S(n)\right|=\sqrt{R^{2}(n)+I^{2}(n)}$$

where *R* and *I* are the real and imaginary parts of the Fourier transform respectively. The magnitude spectrum, *P*(*n*), is then multiplied with Mel filter bands as follows:

(13) $$P_{\text{Mel}}(m)=\sum_{n=0}^{\frac{N}{2}-1}H_{m}(n)P(n)$$

(14) $$H_{m}(n)=\{\begin{array}{c} 0,f(n)<f_{c}(m-1)\\ \frac{f(n)-f_{c}(m-1)}{f_{c}(m)-f_{c}(m-1)},f_{c}(m-1)\leq f(n)\leq f_{c}(m)\\ \frac{f(n)-f_{c}(m+1)}{f_{c}(m)-f_{c}(m+1)},f_{c}(m)\leq f(n)\leq f_{c}(m+1)\\ 0,f_{c}(m+1)\leq f(n) \end{array}$$

(15) $$f_{c}(m)=700\left(10^{\frac{ɛ(m)}{2595}}-1\right)$$

(16) $$ɛ=2595log_{10}\left(\frac{f}{700}+1\right)$$

The MFCCs are finally extracted by applying a discrete cosine transform to *P_Mel_*(*m*):

(17) $$\text{MFCC}(k)=\sum_{m=0}^{M-1}P_{\text{Mel}}(m)\text{cos}\left(\frac{(m+0.5)k\pi }{M}\right)$$

where *M* is the number of Mel filters and *MFCC*(*k*) is the *k^th^* coefficient.

#### 3.2.2. Hidden Markov Model

A hidden Markov model (HMM) is a parametric model that determines the characteristics of data sequences. A HMM parameter set is defined as follows:

(18) Λ={π,A,B}

here *π* is a 1 × *N* vector containing the prior probability distribution of *N* states, *A* is a *N* × *N* transition probability matrix, and *B* is a set of *N* observation density functions. In our case, we directly modeled the continuous input where *B* was defined as:

(19) $$B(i,x)=\sum_{m=1}^{M}\omega_{m}G(x,\mu_{m},\sum_{m})$$

where *i* = 1,2,…,*N* indicates the state index, *M* is the number of Gaussian components, *ω_m_* is the mixing weight of the *m^th^* Gaussian component, and *G*(*x*, *μ_m_*, Σ*_m_*) is a Gaussian density function with mean *μ_m_* and covariance matrix Σ*_m_*.

In the training phase of the HMM, given the input sequence *X* = *x*_1_, *x*_2_, … *x_T_*, the model parameters are updated to maximize the training likelihood *P*(*X*∣Λ). More details about the training algorithm can be found in [23]. After the training phase, each audio class has a corresponding HMM defined by the parameter sets Λ*^Subway^*, Λ*^Bus^*, Λ*^Other^* In the inference phase, given an input sequence *X* = *x*_1_, *x*_2_, … *x_T_*, the likelihood of *X* given a HMM can be computed by:

(20) $$P(X{\;|\;\Lambda }^{C})=\sum_{h_{1},h_{2},\ldots ,h_{T}}\pi (h_{1})B(h_{1},x_{1})\prod_{t=2}^{T}A(h_{t-1},h_{t})B(h_{t},x_{t})$$

where *h_t_* (*t* = 1, 2, …, *T*) is a hidden state value at time *t* and *h_t_* ∈ [1,2, …, *N*]. The likelihood is calculated by using a forward or backward algorithm as described in [23]. Ultimately, the final class label is decided by:

(21) $$\text{Audio Class}=\text{Argma}x_{C\in \{\text{Bus},\text{Subway},\text{Other}\}}P(X\;|\;\Lambda^{C})$$

## 4. Experimental Results

To evaluate our system, we first conducted experiments with the accelerometer and audio classification independently. As described in the previous sections, the proposed system classifies activities into four contexts first, and if the system identifies a ‘transportation’ mode, it starts to collect audio data to determine whether this transportation is via bus or subway. Next, we evaluated an integrated system that combined the accelerometer and audio classifiers. For the evaluation and testing, we collected over 10,000 data samples from 10 volunteer graduate students during a month-long period at various locations. Also for achieving position-free approach, we allowed volunteers to hold a smartphone at anywhere on their body such as attach it on waist, put it in trousers' pocket or just hold it by hands. After collecting sensor data from all volunteers, we categorized them into each activity types based on activity label. Then we constructed activity model of each activity labels—walking, jogging, still, bus (run, jam, stop) and subway (run, stop)—by GMM-based modeling and classification module in the accelerometer classifier. As noted in section 3, the proposed system utilizes sensor data which is collected previous 3 seconds for real-time processing. It means the system does not use previous contexts for recognition processing. The approaches described above enable position-free recognition. We used only the sensors on Android HTC Desire smartphones, Samsung Galaxy S smartphones, and Samsung Galaxy S II smartphones for collecting and recognizing activities.

### 4.1. Accelerometer Classification

**Figure 4.**
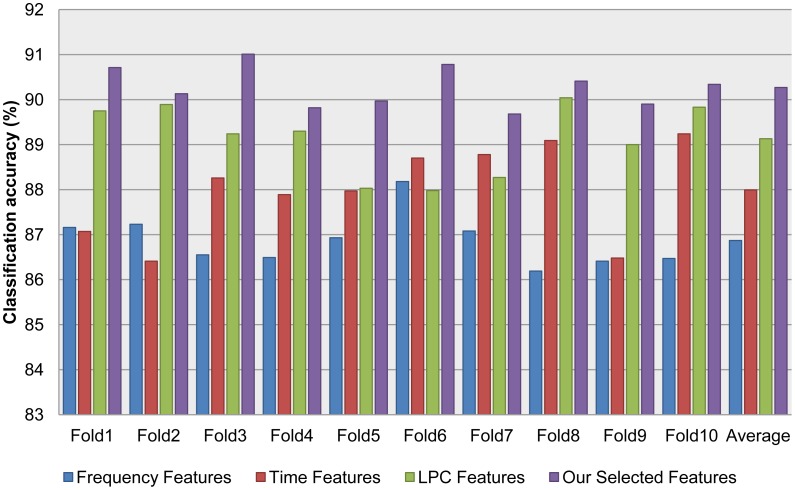
Accelerometer classification accuracy comparison based on Table 1.

In order to validate the accelerometer classification module, we collected acceleration data in four contexts: walking, jogging, transportation (bus and subway), and still, which are available in [24]. As noted in Section 3.1, we evaluated an assortment of features, including frequency, time, and LPC features. To combine the strength of different feature extraction methods, we employed our novel feature selection algorithm to select the best candidate from a large set of features extracted by the existing method. Table 1 and Figure 4 show the 10-fold cross validation test results for different features. Table 2 shows which features were selected from the features generated by the existing feature extraction methods using our proposed feature selection algorithm.

### 4.2. Audio Classification

The dataset we used to evaluate the audio classification was collected and provided by the School of Computing Sciences, University of East Anglia, UK, and is available in [25]. This dataset contained WAV formed audio files (sampling rates: 8 kHz, 8 bit, mono) taken using a Samsung YP55H MP3 recorder in 2004. It had twelve different audio files, but we used seven different contexts: Building Site, Bus, Car (city), Supermarket, Office, Presentation and Street (traffic). Table 3 shows the confusion matrix of the classification measured using a k-fold (k = 10) cross-validation rule.

The average accuracy of our proposed audio classification system was about 97.43%. In addition, we collected our own audio dataset for three contexts—bus, subway, and other (anything except bus and subway)—using various Android smartphones, which is available in [24]. Using a k-fold (k = 10) cross-validation rule, we obtained the accuracy shown in Table 4.

These results present a reasonably high accuracy level, suggesting that audio is an important data source for our context-aware system.

### 4.3. Performance Evaluation of the Integrated System

**Figure 5.**
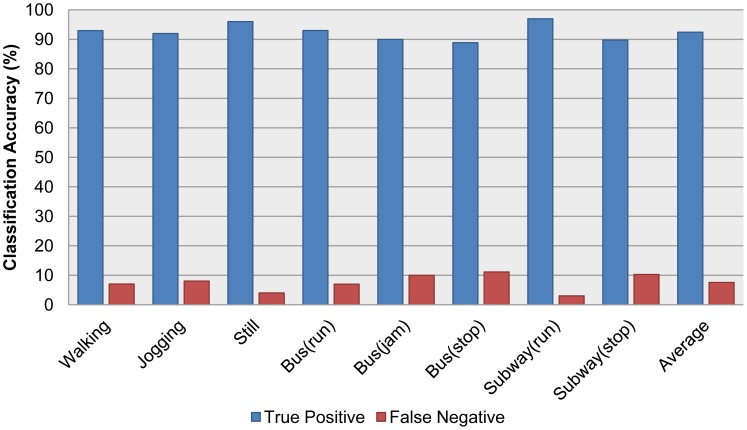
Classification accuracy of the integrated system based on Table 5.

After validating the individual classification module, accelerometer and audio classifiers were combined into one integrated system, with extra information acquired from the GPS and Wi-Fi schemes, as described in Section 3. The integrated system was tested on the field with realistic and real-time sensory data. More specifically, a user launched the system via a smartphone, and as this user moved—e.g., riding a bus or subway—an observer recorded all of the truth labels by hand while the system wrote the recognized labels to a log file. After the test, the recognized labels were compared with the hand-recorded truth tables. The dataset we collected and used for the validation is available in [24]. As described in Table 5 we collected and tested eight different recognizable activities. Three of them are ambulatory activities and the rest of them are transportation activities. Especially riding a bus has another situation ‘Jam’ which might be occurred when a bus is stopped by traffic signal or bad traffic condition. Table 5 shows a confusion matrix of different contexts. We collected over a thousand activities for each context. Figure 5 is a comparison graph of the true positive with the false negative of each activity, which highlights the accuracy of the recognized labels.

## 5. Discussion

The results of the audio classification shows that, by selecting the good features from different feature sets, we can significantly improve the classification accuracy. To validate the significance of the difference between the achievements (when comparing the recognition results of our selected feature set with those of the other feature sets), we used the paired t-test to calculate the p-values, which were always smaller than 0.05 (note that a p-value < 0.05 indicates that the achievements are significantly different from a statistical point of view).

Our experiments clearly show that each individual classifier performed reasonably well, with an average accuracy around 90%. Furthermore, using our proposed feature selection method with the accelerometer classifier was more accurate than using some specific kind of features (p-value < 0.05). By combining the two classifiers with other sensor information, our integrated system successfully recognized different contexts, including not only ambulatory contexts like walking and jogging, but also transportation contexts like the bus and subway. Although the category is still limited by a small number of contexts, we have demonstrated that our multimodal sensor approach has the potential to recognize different kind of contexts. The proposed algorithm for context recognition is mainly focus on how to acquire better classification result by combining accelerometer and audio sensor data. Therefore the accuracy of proposed classification algorithm is presented in Figure 5.

**Figure 6.**
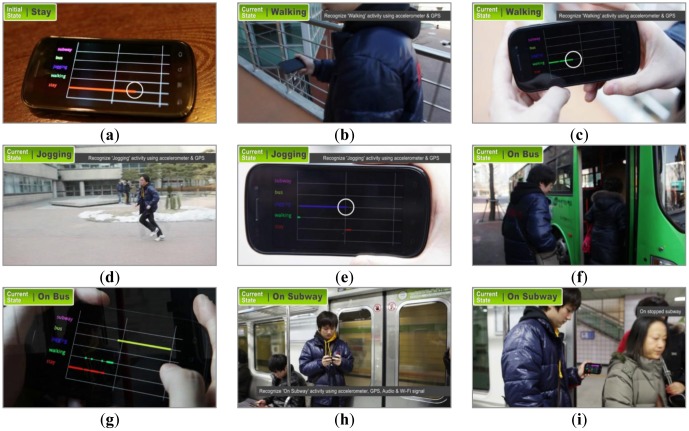
Demonstration of the integrated system via testing in a real-world environment.

In order to test and evaluate the proposed system in the real-world environment, we implemented the system on an Android smartphone as an application. In Figure 6, (a) indicates the initial state of the context recognizer—*i.e.*, ‘Still’—with a red line, (b) shows that the user is walking with the smartphone in his hand, and (c) shows that the application recognized his activity as ‘Walking’ with a green line. When the user started jogging with the smartphone in his pocket, as denoted by (d), the proposed system detected his activity as ‘jogging’ and displayed the movement with a blue line, shown by (e). Subsequently, (f) and (g) show that the user is riding a bus, which is recognized and displayed by the system with a yellow line. The user is riding a subway in (h), which can be recognized even the subway is stopped in (i). A full version of the demonstration movie recorded in real world setting is available on YouTube in [27].

## 6. Conclusions and Future Work

In this work, we have proposed a multimodal approach by utilizing the set of embedded sensors on smartphones in order to recognize different user contexts, such as walking, jogging, riding on a bus, or taking a subway. Overall, we demonstrated that the proposed approach was able to recognize eight contexts, including ambulatory activities and other particular contexts while on a bus or subway. Additionally, it was able to recognize these activities regardless of what the user was doing with his or her smartphone, such as making a phone call, using applications, playing games, or listening to music. Accordingly, we designed and implemented the proposed system, which enabled position-free recognition and was able to recognize activities wherever the smartphone was attached to the body. We also presented a novel algorithm to improve the feature selection phase of the accelerometer classifier, which was shown to increase the recognition accuracy.

Performance evaluations of the accelerometer and audio data classification schemes showed that the proposed algorithm and system performed better than existing approaches. We tested the proposed system by implementing a smartphone application running on an Android OS. These evaluations also showed that the system works well in real-world environments with the accuracy of 92.43%.

Nevertheless, our current system is still limited to a small number of contexts. Further research efforts are necessary to extend the target context category. In addition, the current system is not able to provide detailed information about the recognized contexts, such as bus number, subway line number, or street name while walking. These challenges motivate future research that seeks to utilize other kinds of sensory data to construct a more comprehensive context-aware system.

## Figures and Tables

**Table 1.** Accelerometer classification accuracy with different features.

	**Frequency Features**	**Time Features**	**LPC Features**	**Our Selected Features**
**Fold1**	87.16	87.07	89.75	**90.71**
**Fold2**	87.23	86.41	89.89	**90.13**
**Fold3**	86.55	88.26	89.24	**91.01**
**Fold4**	86.49	87.89	89.30	**89.82**
**Fold5**	86.93	87.97	88.03	**89.97**
**Fold6**	88.18	88.70	87.98	**90.78**
**Fold7**	87.08	88.78	88.27	**89.68**
**Fold8**	86.19	89.09	90.04	**90.41**
**Fold9**	86.41	86.48	89.00	**89.90**
**Fold10**	86.47	89.24	89.83	**90.34**
**Average**	86.87	87.99	89.13	**90.27**

**Table 2.** Selected features from extracted by existing feature extraction methods.

	**Features**	**Selected (X = yes, O = no)**
**Frequency Features**	Over spectral energy	X
	Spectral sub-band 1 energy	X
	Spectral sub-band 2 energy	X
	Spectral sub-band 3 energy	O
	Spectral sub-band 4 energy	O
	Spectral sub-band 5 energy	O
	Spectral sub-band 6 energy	O
	Spectral sub-band 7 energy	O
	Spectral sub-band 8 energy	O

**Linear Predictive Coding (LPC) Features**	LPC coefficient 1	X
	LPC coefficient 2	X
	LPC coefficient 3	O
	LPC coefficient 4	O
	LPC coefficient 5	O
	LPC coefficient 6	X
	LPC estimation error	X

**Time Domain Features**	Mean value	O
	Standard deviation value	X
	Mean crossing rate	X
	XY correlation	X
	YZ correlation	O
	ZX correlation	O

**Table 3.** Accuracy table of audio classification confusion matrix (Ma, L. [26] Dataset).

	**Building Site**	**Bus**	**Car (City)**	**Supermarket**	**Office**	**Presentation**	**Street (Traffic)**	**Total**
**Building Site**	100%	-	-	-	-	-	-	100%
**Bus**	-	100%	-	-	-	-	-	100%
**Car**	-	4%	95%	1%	-	-	-	100%
**Supermarket**	-	-	-	100%	-	-	-	100%
**Office**	-	-	-	-	100%	-	-	100%
**Presentation**	-	-	-	-	-	99%	1%	100%
**Street**	-	-	-	1%	1%	10%	88%	100%

**Table 4.** Accuracy table of audio classification using our dataset [24].

	**Bus**	**Car**	**Other**
**Bus**	89.34%	5.60%	10.66%
**Car**	4.25%	91.20%	4.55%
**Other**	4%	4%	92%

**Table 5.** Evaluation of the integrated system with realistic and real-time data.

	**Ambulatory Activities**			**Bus**			**Subway**		**Total Samples**

	**Walk**	**Jogging**	**Still**	**Run**	**Jam**	**Stop**	**Run**	**Stop**	
**Walk**	1109	36	48	-	-	-	-	-	1193
**Jogging**	25	767	42	-	-	-	-	-	834
**Still**	-	-	1915	-	-	-	20	60	1995
**Bus(run)**	65	86	-	2000	-	-	-	-	2151
**Bus(jam)**	-	-	52	-	782	-	-	35	869
**Bus(stop)**	-	-	16	-	-	279	-	19	314
**Subway(run)**	-	-	24	-	49	-	2341	-	2414
**Subway(stop)**	-	-	18	-	11	7	-	314	350
